# Spinal Regulation of Posture: Effects of Transcutaneous Spinal Cord and Affective Sound Stimulation

**DOI:** 10.3390/life14121569

**Published:** 2024-11-29

**Authors:** Natalia Shamantseva, Varvara Semenova, Olga Timofeeva, Irina Andreeva, Tatiana Moshonkina

**Affiliations:** Pavlov Institute of Physiology, Russian Academy of Sciences, 6 Makarova Enb., 199034 St. Petersburg, Russia; semenovavv@infran.ru (V.S.); ig-andreeva@mail.ru (I.A.); moshonkina@infran.ru (T.M.)

**Keywords:** motor control, vertical posture, spinal cord stimulation, affective sound stimulation, spinal networks, spinal–supraspinal relationship

## Abstract

The combined effects of transcutaneous electrical stimulation (tES) of the spinal cord and affective sound stimulation on postural control were investigated to elucidate the involvement of spinal networks in the maintenance of vertical stability. Healthy volunteers (n = 27) underwent tES and sound stimulation separately and combined quasi-randomly. All participants were field-dependent; i.e., participants used the exteroceptive afferent stream for spatial orientation. Centre-of-pressure parameters were analysed to assess postural stability. Results showed that tES at the T11–T12 vertebrae stabilised posture, tES at the L1–L2 vertebrae had no postural effect, and sound stimulation from the left destabilised posture. To assess the role of spinal regulation of postural disturbances, we compared the effects of combined tES with sound stimulation to those of sound stimulation alone. Stimulation at the T11–T12 level reduced the lateral sway induced by affective sounds, whereas L1–L2 tES did not. These results suggest that, in healthy individuals, spinal networks located at the T11–T12 and L1–L2 vertebral levels have distinct roles in maintaining upright posture, both when a person is standing still and when they are actively stabilising their posture during destabilising perturbations. T11–T12 spinal networks stabilise upright posture when destabilising information is solely transmitted from the supraspinal level.

## 1. Introduction

Postural control requires the continuous integration of visual, vestibular, and somatosensory inputs to address two main tasks: first, the regulation and distribution of tonic muscle activity to maintain the stability of body segments, known as “posture”; and second, the dynamic compensation for both internal and external perturbations, referred to as “balance” [1]. The “posture” task is primarily managed by extensor muscles, which are mainly composed of slow “tonic” fibres, while the “balance” task, including locomotion, is predominantly handled by flexor muscles, consisting of fast fibres [2,3,4].

The spinal and supraspinal networks collaborate to maintain “posture” and “balance”. Spinal mechanisms provide rapid responses to immediate disturbances, whereas supraspinal centres offer more refined control by integrating multiple sensory inputs to adjust posture over longer periods [5,6,7]. N.A. Bernstein captured this constant multisensory interplay with the phrase, “In the organism, all motors are sensored, and all sensors are motored” [8]. However, the question remains as to the role that spinal and supraspinal mechanisms play in maintaining vertical stability during postural destabilisation in humans.

### 1.1. Spinal Networks, Spinal Cord Stimulation, and Postural Regulation

Electrical stimulation of the spinal cord (SC) is effectively used to study body balance control in animal models [9,10]. To study spinal motor control in humans, non-invasive SC electrical stimulation is a smart technique [11]. After the introduction of SC electrical stimulation in research on locomotor and postural responses, it became evident that the role of spinal neural networks in posture regulation is far from clear. Experiments with decerebrated cats demonstrated that tonic epidural electrical stimulation of the SC (L5 segment) enables spinal neural networks to independently manage body weight support without supraspinal input [12]. Similarly, patients with loss of supraspinal regulation, who were unable to stand (severe SC injuries), were able to maintain vertical posture with minimal external support during non-invasive transcutaneous electrical stimulation (tES) of the SC at the T11–T12 or L1–L2 vertebrae [13]. Targeted electrical stimulation at the T11–T12 and L1–L2 vertebrae levels has been shown to recruit different populations of motor neurons through projecting sensory and intraspinal connections, leading to the facilitation of postural synergies [14] and enabling control of leg movements in healthy volunteers [15].

In two of our preliminary studies, we have shown that tES of the lumbar thickening of the SC can significantly modulate “posture”—quiet standing [16,17]. We showed that tES of the SC at the L1–L2 level significantly reduced postural balance by up to 30% by activating predominantly extensor muscles, whereas tES of the SC at the T11–T12 level significantly increased balance by up to 28% by activating predominantly flexor muscles. In both cases, the stimulation effects were manifested when the stimulating electrodes (cathodes) were placed along the midline of the spine or over the left dorsal roots of the SC. The stimulation effect was absent when the cathodes were over the right dorsal roots. We attributed this result to the fact that all participants had a left supporting leg (right dominant leg). There was another nuance. This effect was absent in the participants who used the interoceptive afferent stream for spatial orientation.

### 1.2. Exteroceptive vs. Interoceptive Afferent Stream in Postural Control

The role of sensory information in the neural processes of postural control is being actively investigated, including the influence of afferent information on the spinal mechanisms of postural maintenance [18,19,20].

Previously, people have been divided into groups that use an exteroceptive and an interoceptive afferent stream for spatial orientation; their cognitive styles have been called field dependence (FD) and field independence (FI), respectively [21,22]. Individual cognitive styles influence how a person controls their posture by affecting the processing of sensory information and balance maintenance [23]. Without visual (exteroceptive) flow, in FI subjects, body segments (head, shoulders, pelvis) move independently when maintaining balance. In FD subjects, in the absence of visual information, postural adjustment is achieved by changing the position of the hip joints, and all other body segments (shoulders, head) oscillate “in a block” with the pelvis [24]. We showed that with a restricted exteroceptive afferent stream, when participants stood with their eyes closed in a soundproof chamber, the modulation of spinal network activity affected the posture of the FD participants but not the FI participants [16,17].

### 1.3. Postural Destabilisation by Sound

In recent years, studies have clarified the role of sensory systems and cognitive factors in postural regulation [25,26]. An important yet underexplored aspect of sensorimotor control is the audiomotor control of posture. During human movement, the auditory system provides feedback that assists in adjusting body positions, i.e., audiomotor control, and provides information about changes in the human acoustic environment. These auditory functions occur within the context of active multisensory interaction, in which the postural control system experiences a destabilising effect from the auditory stimuli, particularly when sounds originate from sources that change their localisation in space [27,28]. Affective auditory stimuli can modulate posture depending on their content. It has been shown that negative stimuli have destabilising effects on postural control [29].

### 1.4. Hypothesis and Aims

Audiomotor control of posture is realised with the help of supraspinal levels of postural regulation that involve interplay between multiple brain regions [30,31]. Thus, by applying destabilising sound stimulation, we will influence the spinal centres from the supraspinal level. Most experimental studies of human upright posture control use mechanical perturbation of posture to synchronously activate spinal and supraspinal levels of postural regulation. The combination of tES and destabilising sound stimulation will allow us to elucidate the involvement of spinal mechanisms in the maintenance of vertical stability.

Our previous research has shown that affective stimuli destabilise quiet standing. In this study, we compared the effects of combined tES and similar sound stimulation with those of sound stimulation alone. We looked separately at the involvement of the spinal network that controls the extensor muscles and the network that controls the flexor muscles in postural stabilisation—we stimulated the spinal cord at the L1–L2 and T11–T12 vertebral levels, respectively. This approach allowed us to elucidate how spinal networks regulate posture during periods of postural disturbance.

## 2. Materials and Methods

The study was conducted in accordance with the tenets of the Declaration of Helsinki and approved by the Ethics Committee of the I.M. Sechenov Institute of Evolutionary Physiology and Biochemistry of the Russian Academy of Sciences (Minutes #2-03 dated 26 February 2024). All participants signed an informed consent form.

### 2.1. Participants

Subjects were recruited from students and colleagues who volunteered to participate. They were not aware of the study hypothesis.

The study included 27 volunteers (9 males). The mean age was 26.7 ± 4.5 years. The mean body mass index (BMI) was 21.5 ± 2.2 kg/m^2^. Anthropometric characteristics of the group are shown in Supplementary Materials (Table S1). Exclusion criteria were as follows: age outside the range of 18–35 years, BMI outside the range of 18.5–24.9 kg/m^2^, Gottschaldt test coefficient ≥ 2.5 [32], history of epilepsy, motor and vestibular disorders, or hearing and visual impairment. All participants had normal or corrected-to-normal vision. On the day of the study, the subjects rated themselves as healthy. All had a right dominant leg as determined by the ball kick test. The condition of the auditory system was checked before the study with an AA-02 clinical audiometer (Biomedilen LLC, St. Petersburg, Russia) and a gap detection test. All participants had normal tonal and temporal hearing.

Prior to the study, the cognitive style of the participants was determined using the Group Embedded Figures Test modified by Gottschaldt [32]. Only FD subjects participated in the study because we replicated the experimental environment in which the posture of FI participants was not affected by the tES [16,17]. The flowchart of participant recruitment is presented in Supplementary Materials (Figure S1). Two participants were not recruited because their Gottschaldt test coefficient was equal to 2.5.

### 2.2. Procedure and Tasks

**Figure 1:**
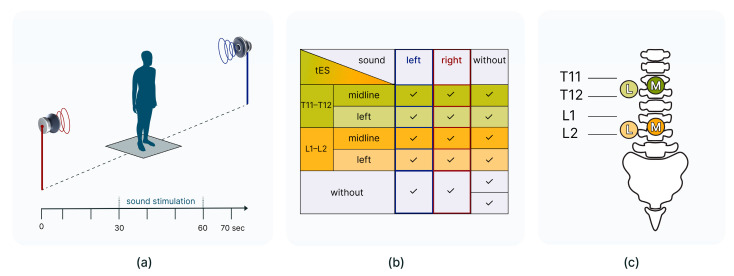
Subject position on a force plate; sound is supplied from left and right speakers (**a**). Variations in spinal cord stimulation and sound stimulation (**b**). Position of cathodes for transcutaneous electrical stimulation of the spinal cord (dorsal view) (**c**).

The study was carried out in the laboratory of the institute. Subjects stood in an anechoic soundproof chamber with their eyes closed on a force plate in a standard posture (heels together, toes apart, arms down along the body). Sound-reproducing speakers were placed to the left and right of the subjects at a distance of 1.5 m from the centre of the interaural axis (Figure 1a).

Two independent sets were used, containing two blocks with four recordings in each (Table 1). Set T11 contained recordings performed with tES at the T11 vertebrae level. Set L1 contained recordings performed with tES at the L1 vertebrae level. Electrical stimulation started a few seconds before the recording and ended a few seconds after the recording. One control recording without spinal and sound stimulation was included in both sets. Overall, for each participant, 16 recordings were made (Figure 1b). Each recording lasted 70 s. After each recording, the subject was allowed to leave the platform for a short rest. A break between two sets lasted for about 30 min. The order of sets and blocks within them was quasi-random.

We did not perform right dorsal root stimulation in the sets because in our previous studies, right dorsal root stimulation did not significantly affect posture [16,17].

In all recordings, participants were provided a cognitive distraction task involving the silent subtraction of a two-digit number from a four-digit number [26]. This trick is usually used in studies of motor control by tSCS when the participants are healthy volunteers to eliminate the possibility of spontaneous effort during the stimulation.

### 2.3. Sound Stimulation

The direction and type of auditory stimulation were chosen based on previous research [33,34]. From the library of the Acoustica Mixcraft audio editor [35], three motivational-affective auditory signals of approximately 8 s duration were selected: ambulance sirens, a car accelerating and braking (sounds of a moving object with negative emotional content), and a mechanical telephone bell (signal with high information uncertainty that regulates everyday behaviour).

The duration of the centre-of-pressure (CoP) trajectory registration was 70 s. The sound signal was provided in the middle of the registration and lasted 30 s. Thus, it was preceded by 30 s of silence and followed by 10 s of silence (Figure 1a).

Sound-reproducing Klipsch R-3800-C speakers (KLIPSCH, Suite, Indianapolis, IN, USA) were used. Sounds were generated from a computer via a Creative E-MU 0202 USB audio interface (Creative Technology Limited, Singapore). The signal level at the speakers was adjusted using a NevaAudio SA-3004 power amplifier (NEVA AUDIO LLC, St. Petersburg, Russia). Signal level measurements at the subject’s head were made using microphone 4145, preamplifier 2639, and amplifier 2606 (all from Brüel and Kjœr, Nærum, Denmark). The intensity of all sound stimuli at the listening position was 79 dB.

### 2.4. Transcutaneous Electrical Stimulation of the Spinal Cord

Location for the tES was selected based on previous studies [16,17]. TES was applied to two zones: at the level of the T11–T12 vertebrae and at the level of the L1–L2 vertebrae (Figure 1c).

Neostim-5 (LTD Cosyma, Moscow, Russia) was used for tES. Stimulation was performed at a frequency of 20 Hz with monopolar modulated current pulses (1 ms, 5 kHz).

Stimulation cathodes at the T11–T12 level (ø 2.5 cm, ValuTrode^®^ Axelgaard Manufacturing Co., Fallbrook, CA, USA) were attached to the skin of the back: one was located along the midline between the spinous processes of the T11–T12 vertebrae (T11 midline), the second was located at a distance of ~1.5 cm to the left and down along the dorsal roots of the spinal cord (T11 left).

L1–L2 stimulation cathodes (ø 2.5 cm, ValuTrode^®^ Axelgaard Manufacturing Co., Fallbrook, CA, USA) were attached to the skin of the back: one was placed in the midline between the spinous processes of the L1–L2 vertebrae (L1 midline), the second was placed at a distance of ~1.5 cm to the left and down along the dorsal roots of the spinal cord (L1 left).

Two anodes (5 × 10 cm^2^, ValuTrode^®^ Axelgaard Manufacturing Co., Fallbrook, CA, USA) were placed symmetrically over the iliac crests.

The intensity of tES was individually adjusted to the maximum level that did not cause pain or discomfort.

### 2.5. Centre-of-Pressure Recording

A Stabilan-01-2 force plate (Rhythm Ltd., Taganrog, Russia) with StabMed 2.13 software was used for CoP recording. The system recorded the CoP positions with a sampling frequency of 50 Hz and a resolution of <0.01 mm.

### 2.6. Centre-of-Pressure Parameters

**Figure 2:**
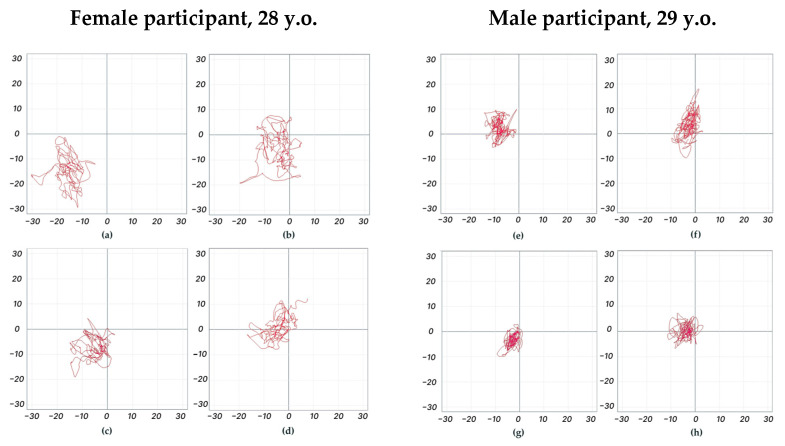
Individual centre-of-pressure trajectories in control without any stimulation (**a**,**e**), with sound stimulation from the left (**b**,**f**), with transcutaneous electrical stimulation of the left dorsal root of the spinal cord at T11–T12 (**c**,**g**), and with transcutaneous electrical stimulation of the left dorsal root of the spinal cord at T11–T12 and sound stimulation from the left (**d**,**h**); the period analysed is 30 s. Horizontal and vertical axes (mm) of the plots corresponds to *frontal* and *sagittal axes* in the CoP parameters analysis, respectively.

The CoP analysed parameters including the length of the CoP trajectory and the linear velocity along the *frontal* and *sagittal axes*. These results provide information about the direction of the postural response. Formulae of the parameters are provided in Table S2. Increased values of these parameters’ relative reference recordings indicate decreased postural control, while decreased parameters’ relative reference indicate increased postural control. Individual CoP trajectories of the two participants are shown in Figure 2.

### 2.7. Data Analysis

Statistical analysis of the CoP parameters was performed using Analyse-it for Microsoft Excel 6.15.4 2024 (Microsoft Office 2021). The Shapiro–Wilk W test was used to determine the data distribution.

Anthropometric data, scores for Gottschaldt test, and tES current values had normal distribution, and values are presented as mean ± standard deviation. All CoP parameters were not distributed normally; therefore, non-parametric statistics were applied, and values are presented as median [1st quartile (Q1), 3rd quartile (Q3)].

The significance of differences between experimental conditions was determined using the Wilcoxon test (differences are presented as *p*-values). The significance threshold was set at the level *p* < 0.05. The effect size (γ_p_) was calculated using the Thompson–Savur method, which is a nonparametric adaptation for quantifying effect sizes analogous to Cohen’s d [36].

## 3. Results

The average score for the Gottschaldt test was 1.6 ± 0.3, which indicates pronounced FD among the participants.

To combine the stimulation results recorded in the T11 and L1 sets of the experimental procedure, we compared the parameters recorded in the controls (without any stimulation) between the sets. The values of all but one CoP parameter, recorded in control in the T11 and L1 sets, were not significantly different. The differences in velocity along the *sagittal axis* recorded in the control tended to be significantly different (*p* = 0.055) (Table 2). For this parameter, we did not combine the stimulation results.

### 3.1. Transcutaneous Electrical Stimulation of the Spinal Cord

Current intensity for tES ranged from 13 to 57 mA (Table 3).

**Figure 3:**
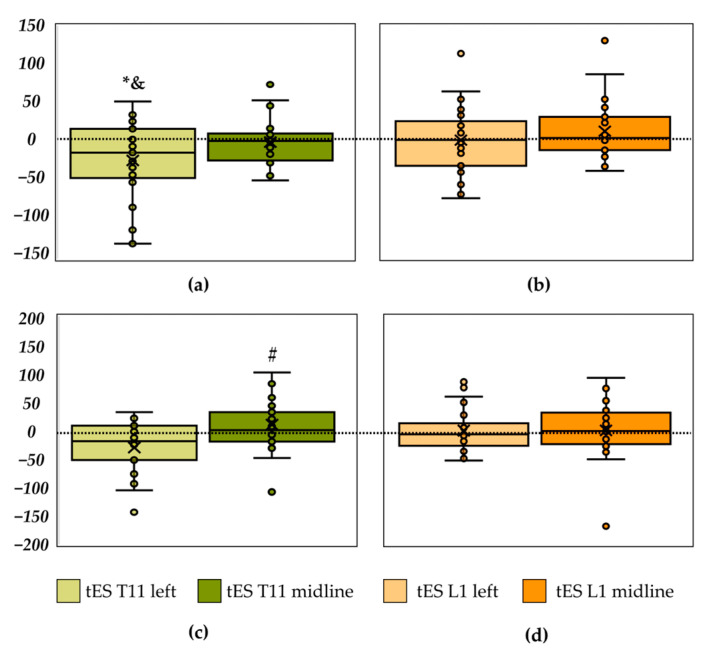
Differences between the length of the centre-of-pressure trajectory along the frontal axis in recordings with the transcutaneous electrical stimulation (tES) and control recording. tES at the T11–T12 vertebrae level (in mm) (**a**); tES at the L1–L2 level (**b**). Differences between the length of the centre-of-pressure trajectory along the sagittal axis in recordings with the tES and control recording (in mm). tES at the T11–T12 vertebrae level (**c**); tES at the L1–L2 level (**d**). * *p* < 0.05; # *p* = 0.05; &—significant difference in the velocity (*p* < 0.05).

Comparison between recordings with tES of the left dorsal root of the SC at the T11–T12 vertebrae level and control showed a significant decrease in the length of the CoP trajectory along the *frontal axis* by 9% (*p* = 0.033) and in velocity along the *frontal axis*—also by 9% (*p* = 0.029) (Figure 3a, Table 4), γ_p_ = 0.83. Midline T11–T12 tES showed a tendency towards an increase by 11% (*p* = 0.051) in the length of the CoP trajectory along the *sagittal axis* compared to control (Figure 3c, Table 4), γ_p_ = 0.53. Thus, stimulation at the T11–T12 level stabilised the vertical posture in the *frontal plane*.

Stimulation at the left side and midline L1–L2 vertebrae level showed no significant differences with control (Figure 3b,d, Table 4).

### 3.2. Sound Stimulation

**Figure 4:**
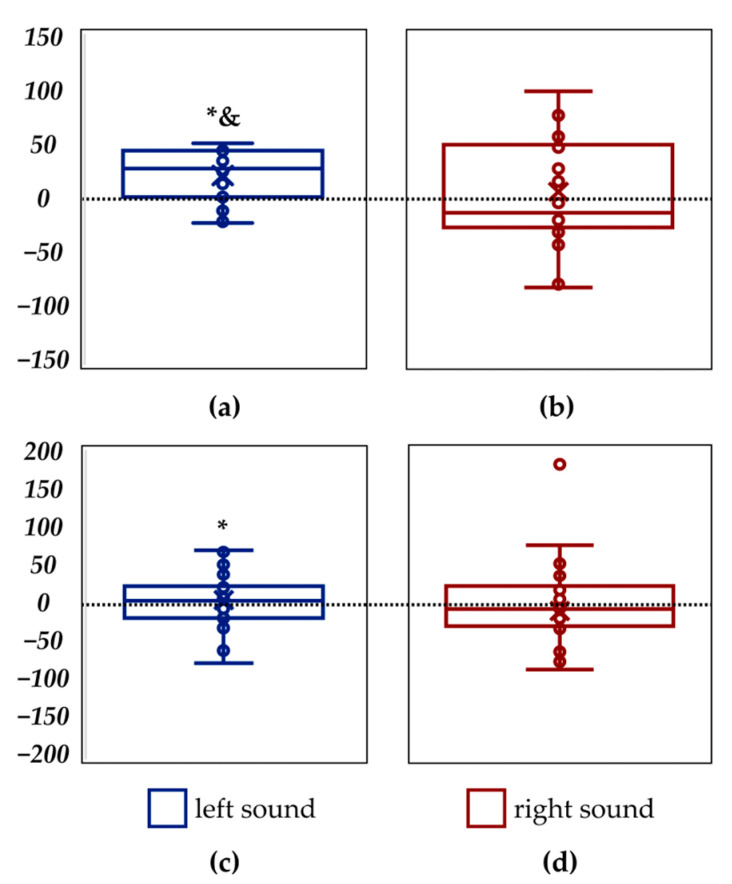
Differences between the length of the centre-of-pressure trajectory along the *frontal axis* in recordings with the sound stimulation and control recording (in mm): left sound (**a**); right sound (**b**). Differences between the length of the centre-of-pressure trajectory along the *sagittal axis* in recordings with the sound stimulation and control recording (in mm): left sound (**c**); right sound (**d**). * *p* < 0.05; &—significant difference in the velocity (*p* < 0.05).

Compared to the control, sound stimulation from the left resulted in a significant increase in length of the CoP trajectory along the *frontal axis* by 13% (*p* = 0.0008) and in velocity along the *frontal axis* by 11% (*p* = 0.0004) (Figure 4a, Table 5), γ_p_ = 0.86. Also, a significant increase, by 10% (*p* = 0.038), occurred in length along the *sagittal axis* during the left sound stimulation compared to control (Figure 4c, Table 5), γ_p_ = 0.58. Thus, left sound stimulation significantly destabilised the vertical posture.

No significant differences were found when the control CoP parameters were compared with the parameters for the right sound stimulation (Figure 4b,d, Table 5).

### 3.3. Combination of Transcutaneous Electrical Stimulation of the Spinal Cord and Sound Stimulation

We compared the effects of combined tES/sound stimulation with the recording of sound stimulation alone to assess the role of spinal regulation induced by tES during periods of postural perturbation.

**Figure 5:**
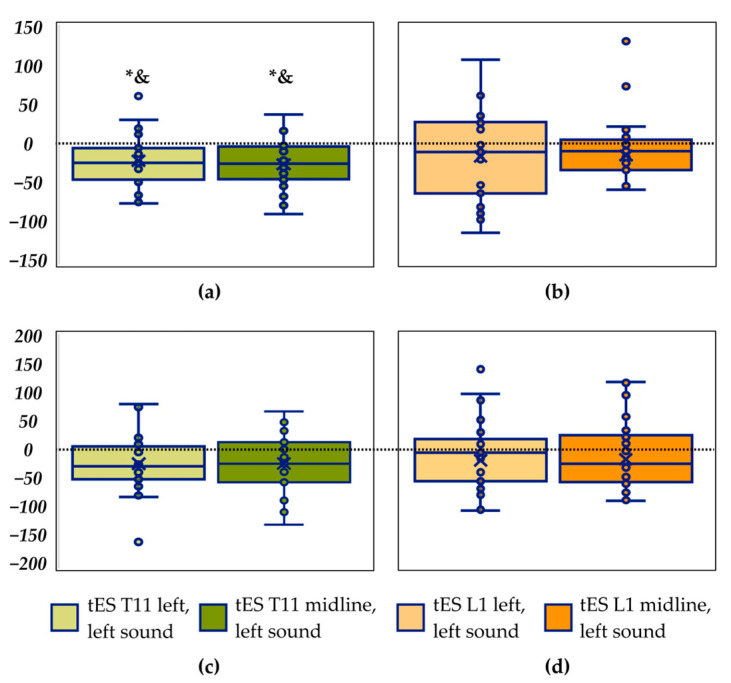
Differences between the length of the centre-of-pressure trajectory along the *frontal axis* in recordings with combined transcutaneous electrical stimulation (tES)/left sound and recording with left sound only (in mm); T11–T12 vertebrae level (**a**); L1–L2 vertebrae level (**b**). Differences between the length of the centre-of-pressure trajectory along the *sagittal axis* in recordings with combined tES/left sound and recording with left sound only (in mm); T11–T12 vertebrae level (**c**); L1–L2 vertebrae level (**d**). * *p* < 0.05; &—significant difference in the velocity (*p* < 0.05).

Compared to the sounds from the left, combined left tES at the T11–T12 vertebrae level with sounds from the left showed a significant decrease in the length of the CoP trajectory and velocity along the *frontal axis* by 11% (*p* = 0.004 and *p* = 0.004, respectively) (Figure 5a, Table 6), γ_p_ = 0.86. Compared to the sounds from the left, combined midline tES at the T11–T12 level with the left side sounds showed a significant decrease in the trajectory length and velocity along the *frontal axis* by 14% (*p* = 0.001 and *p* = 0.001, respectively) (Figure 5a, Table 6), γ_p_ = 0.90. No differences were found for the combination of L1–L2 tES with left sounds in the trajectory length along the *frontal axis* (Figure 5b, Table 6). Along the *sagittal axis,* none of the CoP parameters recorded in combination of all tES with left sounds showed significant differences from those recorded with left sounds alone (Figure 5c,d, Table 6).

**Figure 6:**
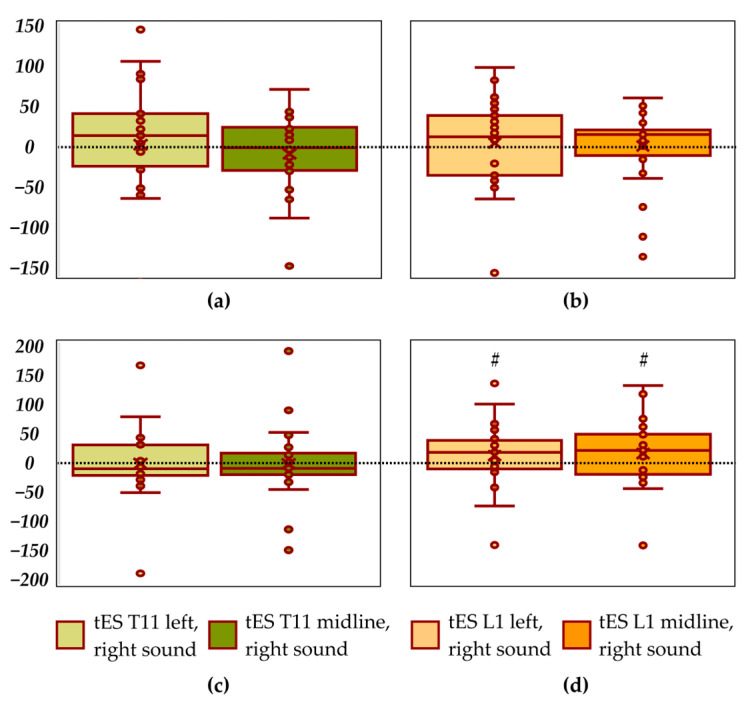
Differences between the length of the centre-of-pressure trajectory along the *frontal axis* in recordings with combined transcutaneous electrical stimulation (tES)/right sound and recording with right sound only (in mm); T11–T12 vertebrae level (**a**); L1–L2 vertebrae level (**b**). Differences between the length of the centre-of-pressure trajectory along the *sagittal axis* in recordings with combined tES/right sound and recording with right sound only (in mm); T11–T12 vertebrae level (**c**); L1–L2 vertebrae level (**d**)*. ^#^ p* < 0.09.

Compared to the sounds from the right, the combination of left and midline L1–L2 tES with sound from the right showed a tendency towards increase by 7% and 6% (*p* = 0.083 and *p* = 0.057, respectively) in the length along the *sagittal axis* (Figure 6d, Table 6), γ_p_ = 0.26 and γ_p_ = 0.21. No difference for this combination occurred along the *frontal axis* (Figure 6b). No significant differences were obtained for combined tES at the T11–T12 vertebrae level and sounds from the right compared to sounds from the right alone (Figure 6a,c, Table 6).

## 4. Discussion

Previous studies have shown that in the absence of distant sensory information, people with different cognitive styles can exhibit high individual variability due to different postural strategies [33,34,37,38], and their posture responded differently to the tES during steady standing [16,17]. In our study, all 27 participants had a pronounced field-dependency coefficient (strictly lower than 2.5). We assume that they showed similar postural responses to sound stimulation and tES.

### 4.1. Transcutaneous Electrical Stimulation of the Spinal Cord

Current intensities in this study ranged from 13 to 57 mA and were consistent with the ranges in our preliminary studies (12 to 55 mA for T11 tES and 10 to 57 mA for L1 tES) [16,17].

Our previous studies have highlighted the significance of considering the cognitive style not only in audiomotor research but also in postural research with tES of the SC [16,17]. In both studies, subjects had no visual and audio information, and only FD subjects’ quiet standing was significantly modulated by the segment-specific tES. We have shown that tES of the left dorsal root at the T11–T12 vertebrae level significantly stabilised postural balance by reducing the length of the CoP trajectory and velocity along the *sagittal axis* in FD participants [17]. We attributed this effect to the activation of the hip flexors. This led to a stiffening of the hip–shoulder unit in the FD subjects. In the absence of a visual frame of reference, FD subjects have shown an increased efficiency of the hip stabilisation in space strategy and an “in a bloc” operation of the shoulder–hip unit [24]. The last “in a bloc” operation extends to the whole head–trunk unit in the absence of visual information [39].

The results of the current study correspond to our previous research on the stabilising effect of spinal stimulation, but the planes of stabilisation are different. In [17], tES of the left dorsal root of the SC at the T11–T12 vertebrae level reduced anteroposterior sway. In this study, left T11 tES resulted in reduced lateral sway (Figure 3a). We hypothesise that the difference in the plane of postural modulation is related to the study protocol and position of the sound sources. The participants did not hear any sounds during the tES recordings under discussion, but they were aware that sound stimulation could be present in any recording. The participants expected the sound stimulation to come from the left or the right in a recording.

In our earlier studies involving tES, all postural recordings were conducted in silence, and the subjects were informed that there would be no sounds. In contrast, this study introduced periodic auditory stimulation, and subjects were unaware of the exact timing of sound delivery and were anticipating the sound in all of the 16 recordings. It has been shown that the anticipation of sound entails audiomotor processes irrespective of its emotional valence and is associated with enhanced activity in the anterior insula, premotor regions, and posterior cerebellum [31]. The posterior cerebellum provides prospective signals about the timing of perceptual events [40] and is involved in anticipatory postural adjustments [41].

It has been shown that FD subjects exhibit anticipatory postural adjustments when expecting auditory stimuli, swaying in the direction opposite to the sound source [37]. In the current study, with sound sources located on both the left and right sides of the participants, these adjustments resulted in lateral anticipatory sway. Thus, tES at the T11 level decreased lateral sway, suggesting an influence on supraspinal mechanisms through the modulation of spinal locomotor networks.

There were no significant differences between results of tES at the L1–L2 vertebrae level and control values (Figure 3). A previous study, that was conducted without auditory stimulation, has shown that both midline and left tES at the L1–L2 level destabilised posture in the frontal plane [16]. We attribute the difference between these studies to a different experimental pre-setting—with and without anticipation of the auditory stimulation.

Based on the spinal stimulation results obtained in the current study, it can be speculated that tES modulates the anticipatory postural adjustments through spinal pathways and facilitates the integration of sensory information at the supraspinal level. This is supported by research indicating that anticipatory postural adjustments are crucial for postural control and are influenced by the central nervous system’s ability to anticipate and respond to perturbations [42,43].

In the present study, the effect of stimulation at the T11 level on posture is not the same as at the L1 level. This confirms the conclusions of our previous studies [16,17] about the different role of neural networks located at these spinal levels in the regulation of quiet standing.

### 4.2. Auditory Stimulation

The main destabilising effect occurred along the *frontal axis* with sound stimulation from the left. Both the length of the CoP trajectory and the velocity along the *frontal* and *sagittal* axes increased significantly, indicating a postural sway, rather than a freezing reaction, where the opposite changes should have occurred—a decrease in the length of the CoP trajectory and velocity [44].

Affective auditory stimuli can modulate posture depending on their content. It has been shown that negative stimuli have a destabilising effect on postural balance [29]. In the current study, we used four affective auditory stimuli—three with negative emotional content and one neutral with high information uncertainty—based on the results of emotional evaluations of audio signals in previous work [34].

Previously, we have investigated the effects of different types of auditory affective stimuli on quiet standing, using long signals without pauses, long signals with pauses, and short signals with pauses coming from all four directions [33]. Based on these results, we have chosen to use three long signals without pauses from speakers located in the *frontal plane* for the current study. We chose long sound stimuli that lasted 8 s, knowing that significant disturbances requiring whole-body adjustments can take up to several seconds to fully stabilise, especially when multiple sensory systems are involved [45]. This correlates with studies of postural control in which the sound stimulation was organised with predominantly long or rhythmic stimuli [27,46,47].

In the current study, we observed a more pronounced destabilising effect of left auditory stimulation predominantly along the *frontal axis*. This effect is probably related to the postural strategy of FD subjects that was described in the previous study [37]. It has been shown that FD and FI subjects use different postural strategies, similar to “fight-or-flight” response, when anticipating and responding to auditory stimuli. FD participants show a postural stabilisation immediately before the sound stimuli but show a significant shift away from the sound source upon presentation—the so-called “flight” response.

No significant changes were obtained with right auditory stimulation, although, on the basis of the previous work [33], we assumed that there would be such an effect. In our previous study with 33 (3 men) young participants (age 18–24), sounds from the right significantly destabilised the quiet standing posture. However, there was no distinction based on the cognitive type of the participants; therefore, this significant factor was not included in our previous research. We hypothesise that the outcome in the present study is related to the participants’ pronounced field dependency and older age.

### 4.3. Combined Stimulation: Spinal and Supraspinal Levels of Postural Regulation

Both midline tES and tES of the left roots of the SC at the T11–T12 vertebral level, combined with sounds from the left, resulted in a significant decrease in the length of the CoP trajectory and velocity along the *frontal axis* compared to sound stimulation alone (Figure 5a). That is, subjects had less lateral sway induced by affective sounds. Thus, tES at the T11 vertebral level offsets the destabilising effect of affective sound stimulation.

Combined tES of the left roots of the SC and midline at the L1–L2 vertebrae level with sounds from the right showed a tendency to increase sway along the *sagittal axis* compared to sounds from the right alone (Figure 6d).

Thus, the effect of stimulation at the T11 vertebral level on posture destabilised by affective sounds was not the same as at the L1 vertebral level. This result demonstrates the different functions of neural networks located at the corresponding levels of the spinal cord in maintaining vertical balance under destabilising external stimuli. The accumulation of numerous studies shows different functions of these vertebral levels in the control of locomotion: at the level of the T11–T12 vertebrae, corresponding to the L2–L3 spinal cord segments, there are neural networks that coordinate the activity of the flexor muscles, initiating locomotion (modulated swing stepping phase); and at the level of the L1–L2 vertebrae, corresponding to the L4–L5 spinal cord segments, there are networks that coordinate the activity of the extensor muscles (modulated stance phase) [11,15,48]. The present study is the first to show the different roles of these spinal levels in maintaining upright posture in normal healthy humans.

Using affective sounds to destabilise upright posture, we influenced spinal centres of posture regulation from the supraspinal level. Previous studies of the spinal networks responsible for posture regulation either excluded the supraspinal level or did not separate the spinal and supraspinal levels when destabilising posture.

The study by Musienko and colleagues [12] showed the role of spinal networks at the level of the L5 segment in body weight support. The research was carried out using the method of epidural electrical stimulation of the spinal cord in decerebrated cats. The animals were able to maintain their body balance when the treadmill belt was tilted. Decerebration was used specifically to eliminate vestibular, visual, and any supraspinal sensory input. Omofuma and colleagues [49] studied the spinal regulation of vertical posture by stimulating the spinal cord transcutaneously, midline between the L2–L3 vertebrae, simultaneous with a balance test. The posture was destabilised by perturbing the participants at the trunk while they stood upright. The immediate effect of tES was to increase leg muscle activity during forward perturbation, but this was accompanied by reduced balance performance in that direction. This mechanical destabilisation of posture modulates the activity of both spinal and supraspinal levels of postural control.

Thus, based on the results of our study, we suggest that spinal neural networks at the level of the T11–T12 vertebrae are critical in stabilising upright posture under a destabilising influence signalled from the supraspinal level and that spinal networks at the level of the L1–L2 vertebrae are unlikely to be involved in this regulation.

## 5. Limitations and Future Directions

The statistical significance of the results was confirmed using non-parametric methods, which are less powerful than parametric methods. We conducted an exploratory study and could not know in advance the sample size needed to use parametric statistics. It will be beneficial to confirm the results with a larger number of participants.

Conclusions about the role of spinal networks in postural regulation and about the interaction between spinal and supraspinal levels of postural regulation are based on the results obtained from studies of a specific group of participants. The study involved field-dependent subjects, i.e., those who used the exteroceptive afferent stream for spatial orientation. We expect that a different research protocol and/or different methods of recording movement and posture in a study with field-independent participants will confirm these conclusions. We did not use electromyography due to the signal cancellation in the anechoic chamber. We see potential in simultaneous recording of postural balance and optical motion capture for detailed analysis of postural strategy based on joint coordination.

## 6. Conclusions

Our experimental findings demonstrate that spinal networks at the T11–T12 and L1–L2 vertebral levels in healthy individuals have distinct roles in maintaining upright posture. These networks function differently when a person is standing still compared to when they are actively stabilising their posture during destabilising perturbations. T11–T12 spinal networks stabilise upright posture when destabilising information is solely transmitted from the supraspinal level. Further studies with simultaneous recording of the centre-of-pressure trajectory, muscle activity, and joint movements are needed to test these findings in the subjects using predominantly the interoceptive afferent stream for spatial orientation.

## Figures and Tables

**Table 1:** Experimental sets and blocks.

T11 Set		L1 Set	
Block 1	Block 2	Block 1	Block 2
tES midline T11	tES midline T11/left sound	tES midline L1	tES L1 left/left sound
Left sound	tES T11 left	tES midline L1/left sound	Right sound
tES T11 left/right sound	tES midline T11/right sound	Control	tES midline L1/right sound
Control	tES T11 left/left sound	tES L1 left/right sound	tES L1 left

**Table 2:** Control CoP parameters in T11 and L1 sets of the experimental procedure.

***Frontal Axis***				***Sagittal Axis***			
Length (mm)		Velocity (mm/sec)		Length (mm)		Velocity (mm/sec)	
T11 Set	L1 Set	T11 Set	L1 Set	T11 Set	L1 Set	T11 Set	L1 Set
158 [123;230]	158 [113;205]	5.2 [4;7.5]	5.2 [3.7;6.7]	222 [165;290]	207 [144;268]	7.3 [5.3;9.4]	6.6 [4.7;8.8] ^#^

^#^*p* = 0.055; comparison between the similar values obtained in the T11 set and the L1 set.

**Table 3:** Current intensities of the tES in mA.

T11 Left Dorsal Roots	T11 Midline	L1 Left Dorsal Roots	L1 Midline
34 ± 8	31 ± 8	32 ± 8	28 ± 8

**Table 4:** Length of the centre-of-pressure trajectory and velocity along the *frontal* and *sagittal axes* in recordings with transcutaneous electrical stimulation at the left side and midline T11–T12 and L1–L2 vertebrae levels.

***Frontal Axis***							
Length (mm)				Velocity (mm/sec)			
tES T11 Left	tES T11 Midline	tES L1 Left	tES L1 Midline	tES T11 Left	tES T11 Midline	tES L1 Left	tES L1 Midline
140 [111;207] *	153 [116;192]	159 [113;194]	148 [115;201]	4.6 [3.6;6.8] *	4.9 [3.7;6.2]	5.2 [3.6;6.4]	4.7 [3.8;6.6]
***Sagittal axis***							
209 [153;256]	215 [172;289] ^#^	223 [166;271]	210 [151;285]				

* *p* < 0.05; **^#^***p* = 0.05; comparison with the control values shown in Table 2.

**Table 5:** Length of the CoP trajectory and velocity along the *frontal* and *sagittal axes* in recordings with sound stimulation.

***Frontal Axis***			
Length (mm)		Velocity (mm/sec)	
Left Sound	Right Sound	Left Sound	Right Sound
173 [145;243] *	154 [106;203]	5.7 [4.8;7.9] *	5.1 [3.5;6.5]
***Sagittal axis***			
216 [170;289] *	202 [158;263]		

* *p* < 0.05; comparison with the control values shown in Table 2.

**Table 6:** Length of the CoP trajectory and velocity along the *frontal* and *sagittal axes* in combined conditions with transcutaneous electrical stimulation and sounds.

***Frontal Axis***							
Length (mm)				Velocity (mm/sec)			
tES T11 Left	tES T11 Midline	tES L1 Left	tES L1 Midline	tES T11 Left	tES T11 Midline	tES L1 Left	tES L1 Midline
**Left sound**							
159 [105;198] *	140 [113;190] *	181 [122;207]	163 [125;216]	5.2 [3.4;6.5] *	4.4 [3.7;6.1] *	5.9 [4.0;6.6]	5.3 [4.0;6.9]
**Right sound**							
158 [129;211]	139 [114;243]	172 [121;217]	182 [126;211]	5.2 [4.2;6.8]	4.5 [3.6;7.0]	5.6 [3.9;7.1]	5.9 [4.1;6.8]
***Sagittal axis***							
**Left sound**							
224 [147;259]	208 [156;275]	210 [174;279]	233 [149;278]				
**Right sound**							
201 [169;284]	208 [156;275]	206 [168;320] ^#^	235 [181;299] ^#^				

* *p* < 0.01; ^#^ 0.05 ≤ *p* < 0.09; relative to similar sound stimulation, Table 5.

## Data Availability

The datasets analysed in the current study are available from the corresponding author upon reasonable request.

## Supplementary Materials

The following supporting information can be downloaded at: https://www.mdpi.com/article/10.3390/life14121569/s1, Table S1. Demographic and anthropometric characteristics of the participants; Figure S1. Flowchart of participants recruitment; Table S2. Analysed centre-of-pressure (CoP) parameters.
