# Supplementary material for: Spinal Regulation of Posture: Effects of Transcutaneous Spinal Cord and Affective Sound Stimulation
Source: Life (Basel). 2024 Nov 29;14(12):1569. doi: 10.3390/life14121569 (PMC11676152; doi:10.3390/life14121569)
Supplement: Supplementary file 1 [file life-14-01569-s001.zip › life-3285331-supplementary.pdf]

Table S1. Demographic and anthropometric characteristics of the participants

**n**		**27**
Gender, n (%)	Female	18 (67%)
	Male	9 (33%)
Age, years	Female	25.2 ± 4.9
	Male	29.1 ± 3.9
Height, m	Female	1.65 ± 0.04
	Male	1.80 ± 0.04
Weight, kg	Female	56.6 ± 6.6
	Male	78.8 ± 4.8
Body Mass Index, kg/m^2	All	21.5 ± 2.2

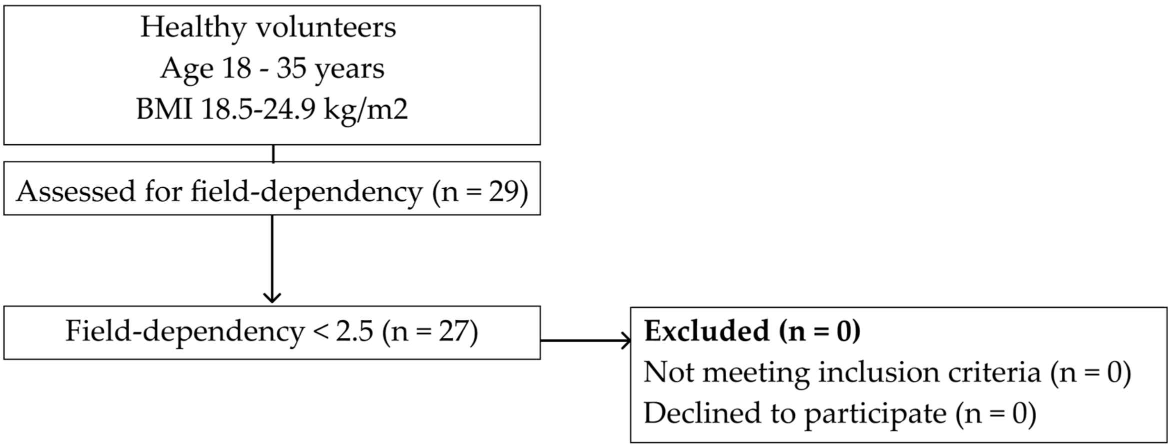

Figure S1. Flowchart of participants recruitment.

Table S2. Analysed centre-of-pressure (CoP) parameters

Parameter	Definition	Formula
Length of the CoP trajectory along the frontal axis	Length of the frontal component of the CoP signal	$LX = \sum_{i=1}^{N}(X_i - X_{i-1})$
Length of the CoP trajectory along the sagittal axis	Length of the sagittal component of the CoP signal	$LY = \sum_{i=1}^{N}(Y_i - Y_{i-1})$
Linear velocity along the frontal axis	The average projection of the linear velocity in the frontal plane	$Vx_{cp} = \frac{1}{N}\sum_{i=1}^{N}\frac{Vx_i}{T}$
Linear velocity along the sagittal axis	The average projection of the linear velocity in the sagittal plane	$Vy_{cp} = \frac{1}{N}\sum_{i=1}^{N}\frac{Vy_i}{T}$

X_i. Y_i – CoP coordinates in time

N – number of counts
